# Neonatal Handling Affects Durably Bonding and Social Development

**DOI:** 10.1371/journal.pone.0005216

**Published:** 2009-04-08

**Authors:** Séverine Henry, Marie-Annick Richard-Yris, Sylvie Tordjman, Martine Hausberger

**Affiliations:** 1 UMR CNRS 6552 Ethos, Ethologie animale et humaine, Université de Rennes 1, Rennes, France; 2 UMR CNRS 6552 Ethos, Ethologie animale et humaine, Station Biologique de Paimpont, Paimpont, France; 3 Service Hospitalo-Universitaire de Psychiatrie de l'Enfant et de l'Adolescent, Centre Hospitalier Guillaume Régnier - Université de Rennes 1, Rennes, France; University of Lethbridge, Canada

## Abstract

The neonatal period in humans and in most mammals is characterized by intense mother-young interactions favoring pair bonding and the adaptation of neonates to their new environment. However, in many post-delivery procedures, human babies commonly experience combined maternal separation and intense handling for about one hour post-birth. Currently, the effects of such disturbances on later attachment and on the development of newborns are still debated: clearly, further investigations are required. As animals present good models for controlled experimentation, we chose domestic horses to investigate this issue. Horses, like humans, are characterized by single births, long lactating periods and selective mother-infant bonds. Routine postnatal procedures for foals, as for human babies, also involve intense handling and maternal separation. In the present study, we monitored the behavior of foals from early stages of development to “adolescence”, in a normal ecological context (social groups with adults and peers). Experimental foals, separated from their mothers and handled for only 1 hour post-birth, were compared to control foals, left undisturbed after birth. Our results revealed short- and long-term effects of this unique neonatal experience on attachment and subsequent social competences. Thus, experimental foals presented patterns of insecure attachment to their mothers (strong dependence on their mothers, little play) and impaired social competences (social withdrawal, aggressiveness) at all ages. We discuss these results in terms of mother-young interactions, timing of interactions and relationships between bonding and subsequent social competences. Our results indicate that this ungulate species could become an interesting animal model. To our knowledge, this is the first clear demonstration that intervention just after birth affects bonding and subsequent social competences (at least until “adolescence”). It opens new research directions for studies on both humans and other animals.

## Introduction

The early post-birth period of humans and of most mammals is characterized by intense mother-young interactions [1]–[2] that help neonates adapt to their novel postnatal environment [3]–[8] and that appear to favor mother-infant bonding [9]–[10]. Although new mothers are considered to play an active part in initiating contacts with their babies [11], newborns also seek contact with their mothers soon after birth. Newborns are also able to regulate their mother's attention as well as the initiation and maintenance of breastfeeding [10], [12], [13]. When human babies are left on their mothers' abdomens during their first hour post-birth, they are able to locate the breast, crawl towards it, grasp a nipple and begin to suckle without any assistance [14]–[16]. However, many maternity routines involve both separation and intensive handling at that precise stage: bathing, weighing, anthropometric measurements, eye ointment, clothing and wrapping in a dry sheet tend to occur before rooming-in with the mother [17] that can sometimes be delayed for up to 4 hours postpartum [18].

Some studies highlight the importance of extended mother-young contacts following birth to obtain optimal pair bonding [19]–[21] and successful breastfeeding [16], [22], but other reports found no beneficial effects [23]–[25]. Therefore, this issue is still highly debated despite of more than 40 epidemiological or clinical studies. More than early mother-neonate contacts, the timing of interactions, as well as the types of handling involved, may be at stake and explain discrepancies between studies. On the one hand, the timing of events set by young in natural situations [1], [15] can be disrupted by neonatal procedures. Studies of postpartum contacts are generally based on the analysis of frequencies of mother - infant contacts without taking into account when they occur [26]. However, Hales et al. [27] mentioned that when prolonged contacts with mothers were delayed for 12 hours, positive effects were reduced compared to when contacts were allowed immediately after birth [26]. Babies kept with their mothers, thus allowing suckling to occur within the first 50 minutes post-birth, were more efficient than those who had been separated from their mothers for 1 hour [22]. According to Jansson et al. [28], separation and handling could interfere with the infant's inborn ability to signal hunger. On the other hand, newborns may be stressed by handling *per se*: according to Long et al. [29], 75% of the hypoxemia events observed in a nursery intensive-care unit were related to handling including suctioning and injections or mere diaper or position changing, physical examinations or feedings. Some authors, like Klaus & Kennell [18], argued recently for an “urgent need to reevaluate routines” and the elimination of unnecessary handling. Whether regulations were still appropriate in 2002 [18] or are now in 2008, remains debatable. Besides, the question of which aspects may be altered by disturbances at this early stage needs to be thoroughly examined.

Most authors in favor of extended contacts report their influence on maternal responsiveness or bonding [26]–[30] and on abandonments [31], [32], but to date few reports give information from the infant's point of view. Some reports indicate, when early contacts did not occur, sleep disturbances, lower body temperatures, higher respiratory rates and higher levels of crying during separation [5], [6], [17], [28], more crying during the first week of life [26] and lower suckling competences [13], [16], [22], [33]. The long-term effects of this early experience (being separated, handled or not, having suckled during their first hour or later) remain unknown, apart from a few indications concerning 3- or 5- year old children [19]. Further investigations are clearly needed to evaluate the importance of the occurrence of maternal separation and handling after birth, as well as the timing of mother-neonate interactions, for the young's behavioral development, especially bonding and, later, attachment. We hypothesized that the stress due to maternal separation at this precise stage (when young should be actively bonding through first feedings) associated with the possible stress of being handled and/or the non-respect of the neonates' “behavioral agenda” could induce insecure attachment and its longer term correlates, such as higher dependency on the mother and lower social competences [34], [35]. Responses concerning humans are difficult to obtain, as later social events and interactions with peers may lower potential effects and longitudinal studies until adulthood last many years. Animal models could yield interesting information, especially as the prenatal and early postnatal stages are the stages when congruence with humans is the highest [36]. Although the pertinence of animal models for developmental studies may be contested, studies of animals remain a source for ideas and research directions [36], as illustrated by the development of the concept of attachment based on early ethological studies [37], [38].

During the last decades, short- and long-term effects of disrupted maternal care have been studied in laboratory rats and non-human primates extensively. Early handling of rats does not induce stressful reactions and can even decrease stress-related behavioral and neuroendocrine responses to stressors in adulthood [39]–[43]. However, longer separations can cause long-term negative effects, as adults can present increased fear-related behavior and accentuated neuroendocrine response to stress [40], [42], [44]. Studies of non-human primates, focusing largely on the effects of the permanent removal of mothers, reveal, among others, long-term deleterious effects on social development, such as inappropriate expression of agonistic interactions, decreased play and social inhibition [45]–[47]. Even a single separation from their mothers (e.g. 6 days) induces robust negative effects [48], [49], the juveniles then present all the features of insecure attachment [50]: they spend less time away from their mothers after being reunited, explore and play less. Data concerning the effects of experience at birth on these privileged animal models are nevertheless relatively scarce. Besides, most animal studies have been made in socially restricted environments [36] and family structure and maternal behavior of females differ greatly between rats and non-human primates (e.g. rats: 8–16 pups per litter and interrupted periods of maternal care; non-human primates: 1 or 2 young at a time and continuous contact with mother; 51, 52).

Female horses, like most primates, have only one young at a time and stay with their young continuously during the early postnatal stages [53]. Bonding occurs rapidly, forming a unique dyadic relation [53] and foals react strongly to short separations from their mothers [54]. Suckling appears to be a major event for the establishment of mother-young bonding for these species [56], [57]. Long-term effects of neonatal and postnatal experience have been evidenced in foals. Handling foals during their neonatal period induces durable mistrust of humans [58], but more interesting still, human interference at first suckling leads to insecure attachment to their mothers and lower social competence when subsequently interacting with peers [59], suggesting, as for humans, a link between quality of attachment and later social competences [34], [35]. Despite differences in developmental and autonomy stages between horses and humans, horses appear to be an interesting animal model to test short- and long-term effects of neonatal maternal separation and handling. Their relatively rapid development allows monitoring of foals from “infancy to adolescence” [60].

Routine neonatal procedures for foals, as for human babies, involving injections, drying and physical examinations, were extended during the last decades under the influence of a veterinarian who promoted the idea of “imprinting” newborn foals [61]. This procedure involves taking the foals away from their mothers (who stay nearby) just after birth and handling them for about an hour. Until now, the possible effects of these routine procedures on the foals' behavioral development are not known. As for babies, they imply a rupture of contact with their mothers, prevent the young from being active and interfere with the “temporal agenda” of bonding. In the present study, we therefore investigated the possible short- and long-term effects of this neonatal procedure on attachment and later social competence of foals maintained in an “ecologically pertinent” environment, namely in paddocks with peers and other mothers.

Our results show that foals submitted to a single 1-hour bout of maternal separation and handling just after birth, compared to controls with no such history, present immediate and delayed behavioral and social disturbances, including insecure attachment to their mothers and lower social competences, lasting at least until prepuberty. This is remarkable as no further handling or separation from their mothers occurred during that period while they lived continuously in their usual social setting. This is to our knowledge the first demonstration showing that interference during the neonatal stage has lasting and profound effects. This finding opens new research directions for both human and animal studies.

## Results

Foals handled when neonates (experimental group) were compared to control foals that were left undisturbed with their mothers after birth. All mares were 5 to 15 years old and all had previous maternal experience. Both groups of foals were observed at different stages, including the neonatal, suckling (when 6 months old), weaning (when 7 months old) and prepuberty stages (when 12 months old). Particular attention was paid to mare-foal distances, exploration and play, as these traits characterize the attachment of young to their mothers [50], [59], [62]. We know that foals tend to initiate distancing from their mothers [63], [64].

We observed no incidence of foal rejection in either of the two groups. No differences between males and females concerning any of the behavioral traits or any stage were evidenced (Mann-Whitney U-test: for all behavioral measures, p>0.05).

### Short-term effects of the neonatal intervention

Control newborn foals left undisturbed with their mothers immediately after birth developed an orderly sequence of behaviors, including glances oriented towards their mothers, first standing up and locomotion, and first suckling. Control neonates first stood up ([16–111] min post-birth) and nursed ([37–161] min post-birth) less than two hours after birth, agreeing with previous reports [65]. The neonatal procedure considerably delayed these first two important developmental stages. Experimental foals (physically separated from their mothers and handled immediately after birth) first stood up ([77–127] min post-birth, Mann-Whitney *U*-test: n _E_ = 9, n _C_ = 10, U = 18, p = .015) and suckled ([123–206] min post-birth, U = 19, p = 0.019) significantly later after birth (table 1). The fact that time between first suckle and first stand up did not differ significantly between experimental foals and controls (U = 42, p = 0.77) suggests a delay in the time course of postnatal events rather than lowered capacities of experimental foals subsequent to handling (table 1).

**Table 1:** Early-handled and control foals' behavioral characteristics from birth to the age of one year.

Stage of development	Measurements	*p*-value	Control	Early-handled
**Neonatal period**	Duration (min) of the handling procedure		**/**	70.2±3.1
	Number of foals with abnormal suckling activities	<.001	[0 out of 9]	[9 out of 10]
	Number of foals with fast-breathing	<.001	[0 out of 9]	[7 out of 10]
	Number of foals with excessive trembling	.01	[0 out of 9]	[5 out of 10]
	Latency (min) to foal first stand	.02	59.4±12.2	101.7±4.5
	Latency (min) to foal first nurse	.02	103.6±12.4	151.2±11.2
	Latency (min) to foal first nurse after first standing	NS	44.2±9.9	49.5±10.4
**Suckling period (6 months**)	Time (%) spent at <1 m from the dam	.01	10.90±1.66	23.78±4.22
	Time (%) spent interacting with the dam	.005	5.20±1.43	8.44±1.47
	Time (%) spent at >10 m from the dam	.02	55.90±11.32	33.11±11.32
	Time (%) spent interacting with same-age peers	.001	7.80±0.91	2.89±1.53
	Time (%) spent in social play activities	.05	4.40±1.28	1.78±1.19
	Number of foals involved in social play	.02	[9 out of 10]	[2 out of 9]
**Prepubertal stage (12 months**)	Time (%) spent at <1 m from same-age peers	.02	74.22±2.64	62.30±3.98
	Mean number of affiliative behaviors per hour	.05	6.78±0.81	5.19±0.73
	Mean number of agonistic behaviors per hour	.08	2.69±0.28	3.88±0.38
	Ratio frequency of affiliative/agonistic behaviors	.05	2.93±0.67	1.40±1.20

a Mann-Whitney U-test.

b Fisher test.

However, experimental foals presented some short-term disturbances, not observed in controls, such as trembling [5 out of 9] (Fisher's exact, p = 0.01), fast-breathing [7 out of 9] (p<0.001) and abnormal sucking patterns prior to nursing [9 out of 9] (p<0.001). Inappropriate suckling patterns included excessive chewing (X̅_E_ = 4.6±1.3 occurrences/hour) and/or teat-seeking (X̅_E_ = 2.7±1.0 occurrences/hour) directed “at the air” or the handler and not towards their mothers. All experimental foals [9 out of 9] also struggled during handling (mean: 11.40±1.49 attempts to stand up) prior to remaining lying motionless with high muscle tone.

### Medium-term effects on mother-young attachment

#### Observations in usual situations

**Figure 1:**
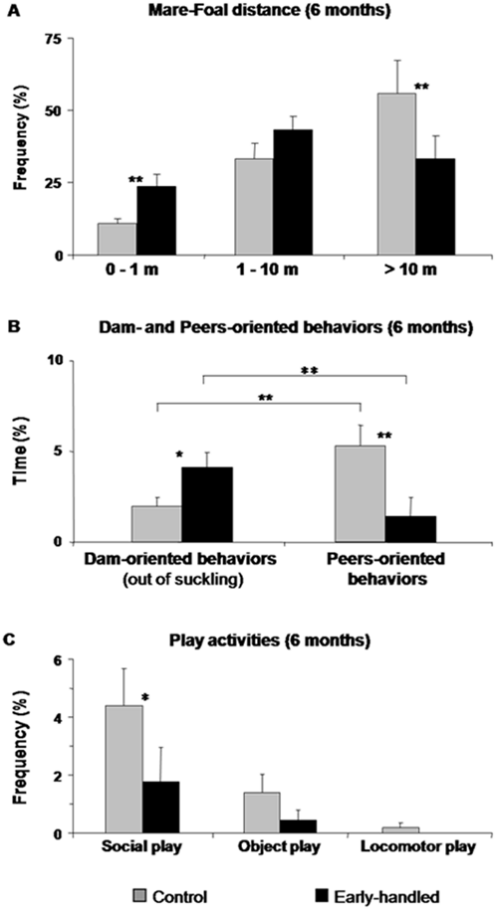
Social behavior of 6-month old early-handled and control foals. A- Time (%) spent at various distances from mother, B- Frequency of mother- and peers-oriented behaviors such as sniffing and mutual grooming, C- Frequency of social and solitary play. Mann-Whitney U-test and Wilcoxon test: * *p*<0.05, ** *p*<0.02, *** *p*<0.005.

When observed at later stages (when 6 months old) in paddocks where they lived with other mare-foal pairs, early-handled foals appeared more depend on their mothers: they kept shorter distances (fig. 1a), interacted preferentially with their mothers rather than with same-age peers (Wilcoxon: t = 1, p = 0.017; fig. 1b, table 1) and played less frequently (U = 20, p = 0.036, table 1), especially social play (fig. 1c). Only 2 of the 9 early-handled foals engaged in social play, whereas almost all control foals [9 out of 10] did (p = 0.017, table 1). In fact, the longer foals remained close to their mothers, the less they played (Spearman correlation: r = −0.87, p = 0.001) or interacted with peers (r = −0.62, p = 0.004). Experimental foals explored even less and more reluctantly in the presence of new “objects” such as an unfamiliar human standing motionless in the paddock: only three of them [3 out of 9] approached and investigated the unfamiliar human, whereas almost all controls [9 out of 10] readily left their mothers and approached the human (Fisher's exact, p = 0.017). Only socio-emotional features seemed to be involved, frequencies of exploration, locomotion, resting standing, urinating, defecating and self-grooming did not differ significantly between early-handled foals and controls (Mann-Whitney *U*-test: n _E_ = 9, n _C_ = 10, for all, p>0.05).

#### Reactions to separation from mother

**Figure 2:**
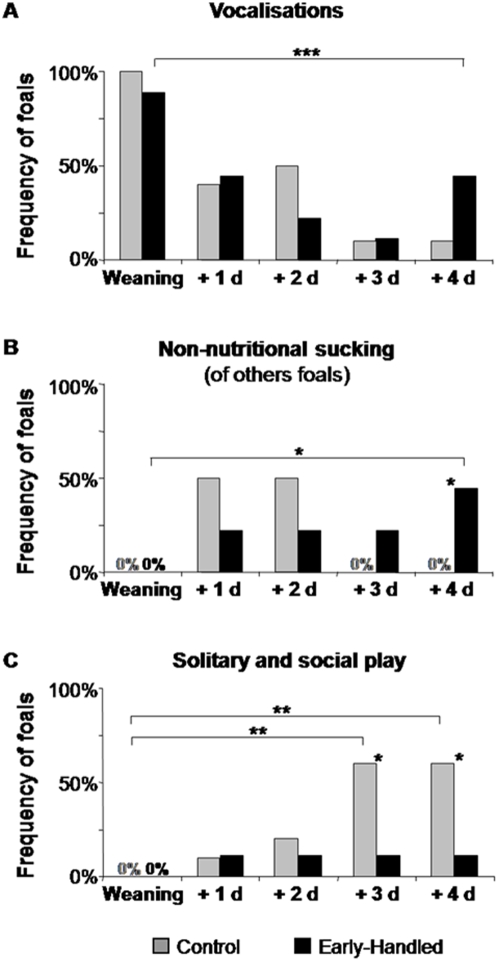
Reactions of 7-month old early-handled and control foals when definitely separated from their mothers (weaning). A- Percentage of foals emitting vocalizations the day of weaning and the four following days, B- Percentage of foals attempting to suckle another foal (non-nutritional sucking), C- Percentage of foals playing. Fisher test: * *p*<0.05, *** *p*<0.001.

When first isolated from their mothers for weaning (when 7 months old), foals of both groups emitted similar levels of vocalizations [a good indicator of social stress; see 54], [65] (X̅_C_ = 14.08±2.80 occurrences/hour; X̅_E_ = 12.96±0.82 occurrences/hour, U = 40, p>0.05) and aggressiveness towards peers (X̅_C_ = 1.72±0.96 occurrences/hour; X̅_E_ = 1.83±0.87 occurrences/hour, U = 33, p>0.05), suggesting apparent similar levels of stress. After the second day post-weaning, the emotional reactions of controls clearly decreased. Conversely, the experimental foals [4 out of 9] continued to display high levels of vocalizations (fig. 2a) and non-nutritional sucking (fig. 2b) even four days after weaning, and fewer of them engaged in either solitary or social play (Fisher test: p = 0.037, fig. 2c).

### Long-term effects on social development

When they were one year old (prepuberty), foals, which have been separated from their mothers 4 months previously, were housed in same-age groups. Most activities, including grazing, locomotion, exploration and resting of foals handled as neonates did not differ significantly from those of controls, but social behavioral profiles appeared to be influenced by early experience. Experimental foals tended to withdraw socially (table 1): they spent significantly more time at greater distances (>1 m) from their same-age peers (X̅_E_ = 38.70±3.98%) than did controls (X̅_C_ = 25.78±2.64, *U* = 20, p = 0.02). More interesting, early-handled foals tended to be more aggressive towards their peers and to interact agonistically more than did control foals (*U* = 23, p = 0.08; table 1). Control foals displayed almost three times more affiliative behaviors than agonistic behaviors (t = 0, p<0.001), but experimental foals did not show this tendency (p>0.05).

## Discussion

Our observations, from infancy to adolescence, of young horses that differed only by their neonatal experience revealed short-, medium- and long-term effects of early experience on attachment and later social competence. Foals that were submitted to a routine procedure consisting in being physically separated from their mothers immediately after birth and handled for a just one 1-hour, presented patterns of insecure attachment to their mothers (strong dependency on their mothers, little exploration or play) and impaired traits of social competence (increased withdrawal and aggressiveness, impaired play) from an early age to young adulthood, while other behavioral features were not affected. These results underline the importance of this early stage for appropriate social development, despite later experience with peers. These findings have general consequences for future research and invite scholars to reconsider post-birth procedures in many domestic mammals, but also in humans.

### Handling stress or reaction to maternal separation?

In the present study, our neonatal procedure involved early handling associated with loss of contact with the mother and, as a consequence, disturbances in the time course of neonatal development, including the first mother-young interactions and pair bonding.

Handling *per se* was obviously stressful for newborn foals, given their strong defense reactions (foals struggled many times) that preceded a period of apparent relaxation (foals remained lying motionless, but presented high muscle tone) that may correspond to a “learned helplessness” [66] as suggested by some authors [67; Sigurjónsdóttir & Gunnarsson, personal comm]. Human neonates appear to experience excessive handling as a stressful event, increasing cortisol levels, levels of crying and grimacing and, in most serious cases, leading to hypoxemia [29], [68], [69]. Most surprisingly is that even simple routine care, like standardized nappy change, may increase heart rate and stress behavioral responses [70], [71]. In addition to these short-term effects, early adverse experiences, in particular during the neonatal period, can cause long-term behavioral and neurobehavioral after-effects, involving higher vulnerability to stress and the development of abnormal behavior [72], [73]. As due to perinatal brain plasticity, early experience, whether adverse or positive, can lead to permanent shifts in the hypothalamic-pituitary-adrenal (HPA) axis, reflecting reactivity to stress [42], [72]–[74].

If the developmental stage at which a stressful event occurs may enhance their degree of severity, being separated from the mother may also enhance their negative impact. The presence of, and contacts with, mothers reduce the stress responses of both human and animal offspring [3], [8], [75]. Various forms of maternal contacts, such as skin-to-skin contact [3], [8], [70], olfactory cues [14] and suckling [72], [76], [77] can attenuate behavioral (distress calls and grimacing) and physiological (heart rate) responses to aversive procedures. Therefore, neonates no doubt experience higher levels of stress when exposed to stressors without their mothers. In addition, enduring highly stressful events without receiving responsive maternal care may alter the development of secure attachment. Considering Bowlby's [42] conceptualization of attachment as a “behavioral safety-regulating system”, several authors [34], [50], [79]–[83] suggested that maternal sensitivity to infant distress could be particularly relevant for the formation of a secure attachment relationship. A reduced amount of maternal behavior subsequently induces higher levels of emotionality, poor play and reduced social competences in non-human primates [84], [85], suggesting insecure attachment [50]. We can therefore question whether lack of maternal care and responsiveness around birth, at the precise period when neonates are expecting intense maternal stimulations [12], [86] and are submitted to “the stress of being born” [20], contributes to the development of attachment insecurity. Therefore, not surprisingly, the present study shows that routine procedures combining lack of maternal responses and stress have long-term effects.

### Impact on mother-young interactions

These procedures prevent neonates from establishing contact with their mothers and from perceiving olfactory cues known to favor their adaptation to the outside world [8], [14], [20]. These events are thought to play important roles in the development of mother-young recognition and bonding in many mammal species (humans: 14, 87; ungulates: 88–90; rats: 91). Maternal separation *per se* is a great source of stress for newborns. The severity of effects may be stronger in highly precocious species (e.g. foals), as bonding naturally occurs very shortly and rapidly after birth, in comparison to less precocious (e.g. human or non-human primates) or highly altricial ones (e.g. rodents). But, even human babies separated from their mothers at birth emit distress cries that decline abruptly when maternal body contact is reinstated [5], [92]. Similarly, exposure to familiar maternal odors or tactual and thermal stimuli reduces levels of locomotor activity and distress calls by isolated neonate animals [93], [94]. Suckling provides comfort and analgesia [10], [76], [77]. In addition, some authors suggest that uninterrupted mother-young contact after birth facilitates first suckling by human infants, and successful first suckling is an important step for pair bonding in several mammal species [17], [56], [57], [95]. Thus the first few suckling sequences are crucial for lambs to develop a mother preference, which is mediated by colostrum [56]. Ingestion of milk activates the endogenous opioid system that plays an important role in supporting associative learning [96]. Suckling appears to be a key event for appropriate bonding in mammals, for instance, the schedule with which rat pups are given milk on one occasion affects their response during the following nursing bout, even 24 hours later [97], suggesting that, in addition, timing of suckling is important.

### The importance of timing

More than early maternal separation, disturbances in the timing of mother-young interactions may explain the deleterious effects it has on attachment. Human assistance at first suckling, by altering the timing normally chosen by newborn foals in natural situations [65], induced suckling difficulties (reluctance to take the nipple) and affected the establishment of secure attachment to their mothers [59]. Our procedure delayed timing of first suckling. Similarly, human newborns, who experienced maternal separation immediately after birth, suckled less efficiently than did newborns left undisturbed with their mothers [25]. Moreover, when forced to suckle instead of being allowed to suckle spontaneously, newborns place their tongues inappropriately around the nipple [33]. Therefore, findings in both humans and foals strongly suggest that letting individuals “set the agenda” [25], [98], [99] and be actors of their own development, especially when crucial events and/or periods (e.g. suckling) are involved, may be a major element ensuring normal behavioral development. In addition, the non-respect of the timing of the first mother-young interactions and suckling may affect mutual adjustment of emotional and attentive states between neonates and their mothers (referred as attunement) that contribute to subsequent attachment security [100], [101].

### Attachment and social competences

Our results indicate that foals handled post-birth and physically separated from their mothers showed not only insecure attachment to their mothers, but also lower levels of social competence despite daily experience with peers. Interestingly, a previous report suggested a similar influence of human interference at first suckling [59], confirming the link between quality of attachment and subsequent social competence in humans and non-human primates [34], [35]. In the present study, early-handled foals remained close to their mothers during their first months and thereby did not develop extensive social relationships with peers. They expressed impaired social skills after their definitive separation from their mothers: they withdrew socially and presented higher levels of aggressiveness towards peers. Under normal conditions (control foals), these two types of relationships emerge sequentially, the first (relationship with mother) subsequently declining over the very same chronological period during which the second (relationships with peers) increases [65]. Each type of relationship provides young foals with specific stimulations that the other either cannot (e.g. nursing) or typically does not (e.g. play with peers). However, as suggested by our results, among others [38], [50], [59], the history of mother-young interactions can influence the set of relationships with peers. Attachment theory predicts that the quality of the mother-child tie has important implications for the social development of a child [38], [50]. Insecurely attached children focus unduly on their mothers, even when external stress is minimal, and consequently interact less with peers compared to securely attached infants that are able to use their mothers as a secure base for exploration [104], [105]. Bowlby [38] suggested that infants with histories of secure attachment developed positive expectations concerning their relationships with others, an inclination to be closely involved with others, as well as social and emotional capacities that promote social competence. Sroufe et al.'s [35] longitudinal study from early childhood to adulthood revealed significant links between secure attachment and social competence (e.g. expectations of relationships, engagement with others, skills in interactions, popularity etc.) at all ages. Our results support these findings and underline the potentialities of our animal model.

### What animal models for developmental research?

Appropriate animal models provide interesting food for thought about human behavior and development concerning long-term effects of early experience on subsequent behavior. Gottlieb and Lickliter [36] argue that experiments on “nonhuman species with behavioral and psychological repertoires that are similar to humans […] may throw light on seemingly related behavior in human beings”. To date, research on the short and long terms effects of early postnatal manipulations of mother-young relationships has been mainly on laboratory rats, as their development is rapid and their mechanisms can be easily studied [106], [107], and to a lesser extent on non-human primates and in particular rhesus monkeys whose maternal behavior, early environment and development present important similarities to those of humans. However, “classical” animal models, although they have proved to be extremely useful, may, for some reasons, be more questionable when behavior and/or ecological contexts are too different.

Mother rats usually give birth to large litters (8–16 altricial pups). They respond maternally to any pup present in their nest and do not seem to recognize individual pups or to form individual bonds [106]. During the first 2 weeks post-birth, intensive maternal care occurs in regular bouts of retrieving, licking and nursing, lasting approximately 1 hour, separated by 15–30 minute periods during which mothers leave their nest and litter [51]. Experimental procedures include daily maternal separation and handling, either for short (15 min/day, referred to as early handling) or long periods (3–6 h/day, referred to as maternal separation). However, most reports focus on effects and responses associated with stressful circumstances and relatively fewer studies address the extent to which these particular neonatal manipulations influence the development of non-stressful behavior, like social behavior [107]. Moreover, the maternal behavior of female rats, with discontinuous periods of maternal care, may explain the low reactivity or even the positive responses of rat pups to short maternal absences and human handling. On the contrary, repeated brief bouts of separation increase foals' sensitivity to maternal separation [e.g. 5*10 min., 55], as for humans and non-human primates [48], [49]. Lastly, the presence of littermates during maternal separation may partly explain the absence of effects on social play frequency in pups submitted to either short or long periods of separation from their mothers [107], conversely to foals and non-human primates [45]–[47].

On the other hand, many studies have investigated the short- and long-term effects of rhesus monkey infant-mother interactions, mostly as a result of the chronic absence of maternal care [108]. Contrary to rodents, non-human primates are relatively well-developed at birth, grow moderately postnatally and their infants are singletons. Mothers provide maternal care (nursing, carrying, licking and grooming) and develop selective bonds with their own young. The early stage of development is usually characterized by continuous mother-infant contact [52]. As for these species loss of maternal contact at an early age is an abnormal situation, maternal deprivation and even short episodes of maternal separation lead to acute long-term disturbances [45]–[49].

To date, very little experimental research both on non-human primates and on rodents has investigated the effects of a single bout of maternal separation and intense handling immediately after birth, at a time when mothers and infants initiate their first contacts [2]. Although most animal studies concern socially restricted environments [36], it is important to consider development in “ecological” contexts that include peers and adults in complex social environments [109]. This was possible in the present study on horses, which, although not classically used as animal models, have enabled us to shed light on, and raise new questions about, the impact of routine post-delivery procedures on human development [59]. Post-delivery routines for foals, as for human babies, involve intense handling, maternal separation and sometimes assistance at first suckling. In addition, horses present many human-like characteristics, since mares give birth to a single young (which implies no interference with littermates), respond selectively to their own newborn and stay with their young continuously during the early postnatal stages [54]. Moreover, although developmental and autonomy stages differ between horses and humans, the precocial state of foals and their short maturation time [65] make them suitable for both longitudinal studies and investigation of filial attachment. Therefore, results of experimental studies based on this animal model could provide important information on the impact, from early age to adulthood, of neonatal experiences.

### Conclusion

This present study, based on a horse model, is, to our knowledge, the first report demonstrating that a short intervention immediately after birth, like a single 1-hour episode of maternal separation and handling, can have effects on the young's behavioral, social and emotional development from birth to at least adolescence. We anticipate our study to be a starting point for important new developments on questions such as the importance of very early first mother-young contacts, the active role played by young in bonding and the biobehavioral substrates of attachment both in humans and animals.

## Materials and Methods

All test procedures and experiments were conducted in accordance with the French regulations governing care and use of research animals.

### Subjects and housing

Subjects were 19 French Saddelbred mares and their foals (*Equus caballus*), 11 females and 8 males, all born at the “Station expérimentale de Chamberet” (France) and all maintained under the same conditions from birth. A few days before parturition, mares were stabled in a 4×4 m foaling stall equipped with a monitoring camera and were observed for signs of parturition every night. Delivery was not assisted and all newborn foals received minimal care apart from the application of an antiseptic on their umbilical stump. During the first five days post-birth, foals spent nights with their mothers in individual box stalls (foaling stall) and were turned out during daylight hours into a pasture with other mares with foals. From the age of 5 days until weaning, all animals were maintained outdoors day and night. Foals were weaned when they were 7 months old and then permanently separated from the mother. They were then kept outdoors for another month. During the winter period (when they were 8–12 months old), they were housed in groups of 6 or 7 foals, in 10 m×50 m pens. From the age of 12 months until the end of the experiment, they returned to 100 m×250 m paddocks and stayed outdoors. Animals were fed with concentrates twice a day when indoors and once a day when outdoors. Water and roughage were available *ad libitum*.

### Experimental groups and procedure

Foals were allocated to one of two treatments on the basis of foaling date, sex and sire: a control group (n = 10; 6 females, 4 males), that included foals and mares left undisturbed after birth and primary care (disinfection of the umbilical stump); and an experimental group (n = 9; 5 females, 4 males) that included foals that had been handled following a routine procedure by an experienced experimenter in the presence of their mothers.

This handling procedure, developed by the veterinarian R. Miller (1991), consisted in handling foals within the 10 minutes following birth, immediately after routine post-natal care (as described above) and before they stood up. During this procedure, foals were restrained and maintained in a recumbent position, while the experimenter stroked them all over their body and exposed them to novel tactile stimuli such as a white towel, a plastic bag and a spray of water. Each stimulus was repeated until the foal remained immobile during the procedure. During the procedure, the mother was in visual contact with her newborn foal, but could not sniff or lick it ad libitum due to human presence. The early handling procedure lasted 72.1±3.4 [52–84] minutes and was performed by a single handler. At the end of the procedure, mothers and foals were left alone. All experimental and control foals were left undisturbed to suckle their mothers. Apart from this early experimental handling procedure, both experimental and control groups received similar limited human contact necessary for routine procedures (mostly feeding and changes of pasture). They were under identical management and groups were mixed at pasture.

### Behavioral observations and assessments: time-budget and social behavior

All foals were observed at four stages: (1) during their early postnatal period, (2) when they were 6 months old and still with their mothers, (3) at weaning, (4) and lastly when they were 1 year old, a prepubertal stage in horses. Observations were video- or tape-recorded and transcribed later. All observations were performed by one observer blind to the observed foal's treatment.

During the immediate postnatal period and when in the foaling stall, the foals' behavior was recorded continuously using focal sampling and 1-minute scans until first suckle was achieved [61]. Particular attention was paid to latencies of the first important developmental stages (latencies to stand up and to suckle) and to potential suckling difficulties, excessive trembling or fast-breathing.

At later stages, foals' behavior was recorded at pasture every 2.5 minutes for 2 hours every day for 4 or 5 consecutive days (200–250 scans per foal) [61]. Observation periods, between 10:00am to 15:00pm, changed every day following a rotation schedule. Each scan recorded the following behavioral items: locomotion, exploration, grazing, feeding, drinking, resting standing, sternal or lateral recumbence, self-grooming and interactions with mother (only prior to weaning) or with other social partners, such as approaches, following, sniffing and mutual grooming. Play patterns were divided into: solitary play (including manipulation of an object and locomotion play) and social play (with mother or peers).

Prior to weaning (when foals were 6 months old), we evaluated mother-foal relationships paying particular attention to mother-foal spatial relationships (contact, 0 to 1 m, 1 to 5 m, 5 to 10 m, 10 to 30 m, >30 m), frequencies of suckling and of other mother-oriented behaviors. At this stage, foals usually explore their environment at some distance from their mothers [30]: they generally spent most of their time (60%) more than 5 m from their mothers, while, for instance, interacting and playing with same-age peers [36], [61]. High levels of exploration play and initiatives to move away from their mothers are assumed to be good indicators of secure attachment to their mothers, in horses as in other mammal species [34], [35].

At weaning (when 7 months old), foals' responses to maternal separation were recorded. Under natural conditions, weaning occurs only when the next foal is born, i.e., when they are around one year old [30], [62]. Early weaning under domestic conditions is currently assumed to be a source of high emotional, physical and physiological stress, mainly due to the abrupt rupture of the mother-foal bond and to the change in housing and feeding practices. Foals generally present strong reactions to their separation from their mothers during the first 48 hours post-weaning [38], [39]. In the present study, foals were weaned in their familiar pasture and were fed with the concentrates and hay that they had previously experienced with their mothers. Foals' reactions were observed on the day they were separated from their mothers and on the following four days.

At a prepubertal stage (12 months old), foals were observed again at pasture, in the presence of their peers. At this prepubertal stage, domestic foals commonly live with same-age peers with whom they may have developed strong social bonds [62], [63]. Particular attention was paid here to foals' distance to their nearest neighbors (contact, 0 to 1 m, 1 to 5 m, 5 to 10 m, 10 to 30 m, >30 m) and to social behavior. Additional focal sampling [60] recorded social interactions for details on frequency (occurrences per hour) and type of social behavior expressed by each yearling: we recorded continuously all affiliative behaviors (social play, initiation to play, following, olfactory investigation and mutual grooming) and agonistic behaviors (threats, bite, kick and chase).

### Test procedure: Reactions to humans

Subjects were tested when they were 6 months old to estimate their reactions to unfamiliar stimuli placed in their environment. Using a classical test [41], [64], we evaluated foals' “willingness” to leave their mothers to explore an unfamiliar human standing motionless 10 m away from the group in the pasture. This test was performed when mares were grazing. We recorded the identity of each foal that moved away from its mother, approached and contacted the experimenter physically (sniffing, licking…). All observations were performed by one experimenter who was blind to the foals' treatment.

### Data analyses

Instantaneously recorded group scans yielded two types of data: (1) percentages of records of different behavioral items (time-budget), and (2) times (in percentage) spent at different distances from mother or nearest neighbor (social proximity). Results are expressed in % (mean±standard deviation). Group continuous recording yielded mean numbers of occurrences of social interactions per hour (social behavior). Results are expressed in frequencies (mean±standard deviation).

### Statistical analyses

As our data did not fit a normal distribution, we used non parametric statistical tests [110]: Mann-Whitney *U*-tests compared two independent samples (e.g. sex differences, early-handled and control foals' time-budget, social behavior and responses to humans); Wilcoxon t-tests compared matched paired data and Spearman tests evaluated correlations. These tests were performed on discrete variables (number of occurrences, latencies) and frequencies, but results are expressed in %. Moreover, Fisher tests on frequencies compared behavioral profiles between groups (presence or absence of behavioral traits). Significance threshold was *p* = 0.05.
